# Crystal structure of hexa­aqua­nickel(II) bis­{5-bromo-7-[(2-hy­droxy­eth­yl)amino]-1-methyl-6-oxido­quinolin-1-ium-3-sulfonate} monohydrate

**DOI:** 10.1107/S2056989016012408

**Published:** 2016-08-05

**Authors:** Hai Le Thi Hong, Vinh Nguyen Thi Ngoc, Anh Do Thi Van, Luc Van Meervelt

**Affiliations:** a Chemistry Department, Hanoi National University of Education, 136 Xuan Thuy, Cau Giay, Hanoi, Vietnam; b KU Leuven – University of Leuven, Department of Chemistry, Celestijnenlaan 200F - bus 2404, B-3001 Heverlee, Belgium

**Keywords:** crystal structure, quinoline, hydrogen bonding, π–π stacking

## Abstract

The asymmetric unit of the title compound, [Ni(H_2_O)_6_](C_12_H_12_BrN_2_O_5_S)_2_·H_2_O, contains a half hexa­aqua­nickel(II) complex cation with the Ni^II^ ion lying on an inversion center, one 5-bromo-7-[(2-hy­droxy­eth­yl)amino]-1-methyl-6-oxido­quinolin-1-ium-3-sulfonate (**QAO**) anion and a half lattice water mol­ecule on a twofold rotation axis. In the crystal, **QAO** anions are stacked in a column along the *c* axis by π–π stacking inter­actions [centroid–centroid distances 3.5922 (10)–3.7223 (11) Å]. The columns are inter­linked by hexa­aqua­nickel(II) cations through O—H⋯O and N—H⋯O hydrogen bonds.

## Abstract 2

The packing of the title compound is built up by columns of π–π stacking quinoline derivatives running along the *c* axis, which are inter­connected by [Ni(H_2_O)_6_]^2+^ complex cations through hydrogen bonding.

## Chemical context   

Among heterocyclic rings, the quinoline ring system is of great importance due to its therapeutic and biological activities. Many new quinoline derivatives have been synthesized and used as new potential agents to treat HIV (Cecchetti *et al.*, 2000 ▸; Tabarrini *et al.*, 2008 ▸) and malaria (Nayyar *et al.*, 2006 ▸) or to inhibit human tumor cell growth (Rashad *et al.*, 2010 ▸). Recently, a simple amino­quinoline derivative has been used in colorimetric sensors for pH (Wang *et al.*, 2014 ▸). In addition, complexes of quinoline compounds with transition metals are also known to exhibit a wide variety of structures and possess profound biochemical activities which allow them to act as anti­microbial, anti-Alzheimer’s (Deraeve *et al.*, 2008 ▸) or anti­tumoral agents (Yan *et al.*, 2012 ▸; Kitanovic *et al.*, 2014 ▸). Some complexes of polysubstituted quinoline compounds have also been used in dye-sensitized solar cells or in efficient organic heterojunction solar cells (Li *et al.*, 2012 ▸).
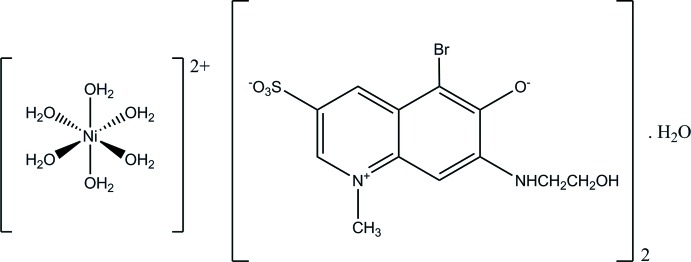


The new quinoline derivative (6-hy­droxy-3-sulfoquinolin-7-yloxy)acetic acid (**Q**) was synthesized from eugenol and its anti­bacterial activities have been reported (Dinh *et al.*, 2012 ▸). From **Q**, a series of polysubstituted quinoline compounds has been synthesized, including 5-bromo-6-hy­droxy-7-[(2-hy­droxy­ethyl)­amino]-1-methyl-3-sulfo­quinoline (**QAO**). As polysubstituted quinoline rings are known to coordinate to metal ions, the reaction between **QAO** and NiCl_2_ was studied. The reaction product could not be characterized unambiguously by IR or ^1^H NMR spectroscopy. Although the obtained spectroscopic data are different from those of free **QAO**, indicating the presence of a deprotonated hydroxyl group, no conclusion about complex formation was possible and further investigation by X-ray diffraction was necessary.

## Structural commentary   

**Figure 1:**
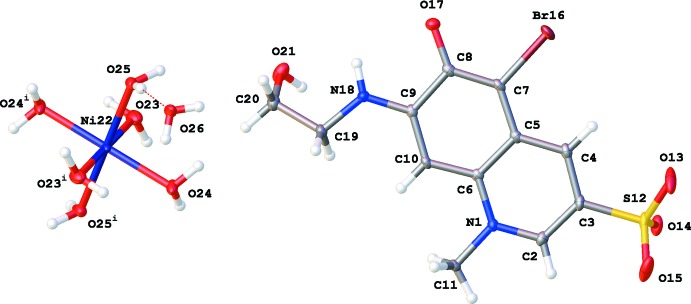
The structures of the mol­ecular components in the title compound with ellipsoids drawn at the 50% probability level. [Symmetry code: (i) −*x* + 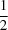, −*y* + 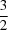, −*z* + 1.]

The structure determination shows that Ni^II^ is not complexed directly with **QAO**, but is present as a hexa­aqua complex, [Ni(H_2_O)_6_]^2+^, located about an inversion center (Fig. 1 ▸). The 6-hy­droxy group as well as the 3-sulfonic acid group of **QAO** are deprotonated. The substituent atom Br16 deviates most [0.125 (1) Å] from the best plane through the quinoline ring system (r.m.s. deviation = 0.009 Å). The 2-hy­droxy­ethyl­amino substituent shows a *+sc* conformation [torsion angle N18—C19—C20—O21 = 57.0 (2)°].

## Supra­molecular features   

**Figure 2:**
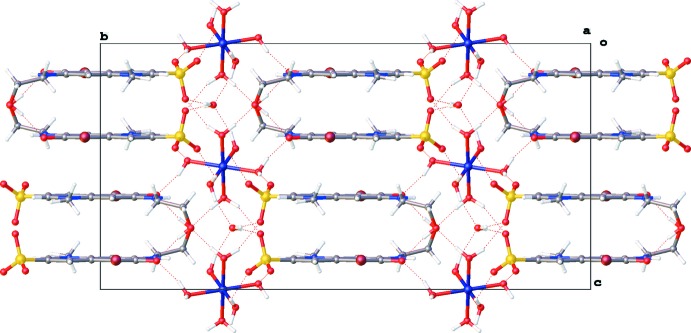
Packing diagram of the title compound viewed along the *a* axis. Dashed lines represent hydrogen bonds.

**Figure 3:**
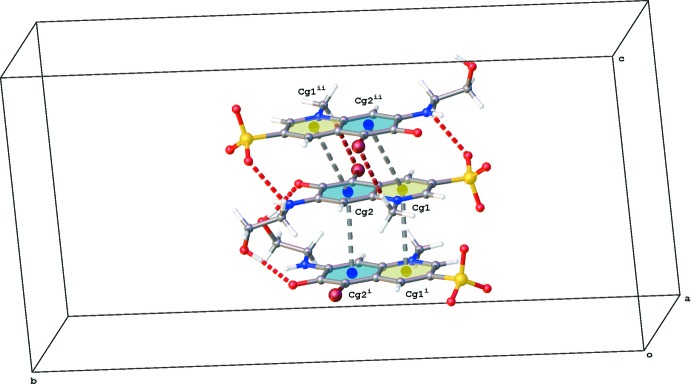
Partial packing diagram of the title compound, showing π–π inter­actions between quinoline ring systems [grey dotted lines; *Cg*1 and *Cg*2 are the centroids of rings N1/C2–C6 and C5–C10, respectively; symmetry codes: (i) −*x* + 2, *y*, −*z* + 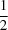; (ii) −*x* + 2, −*y* + 1, −*z* + 1], and C—H⋯Br and C—H⋯O hydrogen bonds (red dotted lines).

**Figure 4:**
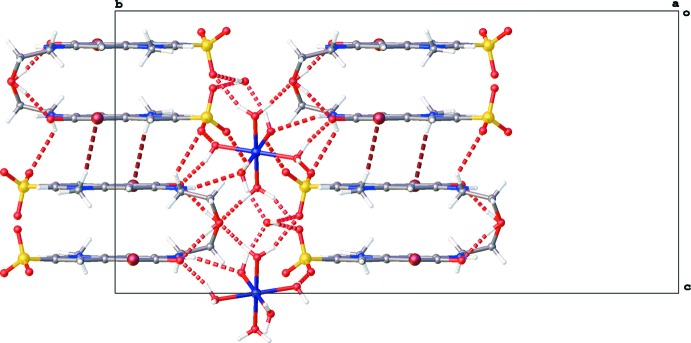
Partial packing diagram of the title compound viewed along the *a* axis, showing the *X*—H⋯O hydrogen bonds (red dotted lines, see Table 1 ▸ for details) and C—H⋯Br inter­actions (brown dotted lines).

The crystal packing (Fig. 2 ▸) is characterized by columns of stacking **QAO** mol­ecules running along the *c* axis through π–π stacking inter­actions between the quinoline ring systems [*Cg*1⋯*Cg*1^i^ = 3.5922 (10) Å, *Cg*2⋯*Cg*2^i^ = 3.5793 (11) Å, *Cg*1⋯*Cg*2^ii^ = 3.7223 (11) Å; *Cg*1 and *Cg*2 are the centroids of the rings N1/C2–C6 and C5–C10, respectively; symmetry codes: (i) −*x* + 2, *y*, −*z* + 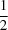; (ii) −*x* + 2, −*y* + 1, −*z* + 1; Fig. 3 ▸]. Within these columns additional C—H⋯Br and C—H⋯O inter­actions occur (Table 1 ▸ and Fig. 3 ▸). The columns inter­act with the hexa­aqua­nickel(II) cations through hydrogen bonding. The lattice water mol­ecule inter­acts with two neighboring cations. One [Ni(H_2_O)_6_]^2+^ complex inter­acts in total with twelve **QAO** mol­ecules and two water mol­ecules through O—H⋯O and N—H⋯O hydrogen bonds (Table 1 ▸ and Fig. 4 ▸).

## Database survey   

A search of the Cambridge Structural Database (Version 5.37; last update May 2016; Groom *et al.*, 2016 ▸) for 3-quinolinium sulfonic acids gives six hits of which four have a zwitterionic form [CSD refcodes PUSMOH (Le Thi Hong *et al.*, 2015 ▸), BAPBOK (Skrzypek & Suwinska, 2002 ▸), HIVHUQ (Skrzypek & Suwinska, 2007 ▸) and QUNREY (Dinh *et al.*, 2012 ▸)]. The remaining two are *N*-methyl­ated [CSD refcode HIVJEC (Skrzypek & Suwinska, 2007 ▸)] or *N*-ethyl­ated [CSD refcode HIVJAY (Skrzypek & Suwinska, 2007 ▸)] and have a hydroxyl group at the 4-position.

## Synthesis and crystallization   

The quinoline derivative (6-hy­droxy-3-sulfoquinolin-7-yloxy)­acetic acid (**Q**) was synthesized starting from the natural product eugenol and further transformed to 5-bromo-6-hy­droxy-7-[(2-hy­droxy­ethyl)­amino]-1-methyl-3-sulfo­quinoline (**QAO**) according to a procedure described by Dinh *et al.* (2012 ▸).

A solution containing NiCl_2_·6H_2_O (262 mg, 1.1 mmol) in 10 mL water was added dropwise to 15 mL aqueous solution of **QAO** (754 mg, 2 mmol) and NH_3_ (pH ≃ 6–7). The obtained solution was stirred and refluxed at 313–323 K for three h. The brown precipitate was collected by filtration, washed consec­utively with ethanol and dried *in vacuo*. The obtained crystals were soluble in water and DMSO, but insoluble in ethanol, acetone and chloro­form. The yield was 60%. Single crystals suitable for X-ray investigation were obtained by slow evaporation from a ethanol–water (1:2 *v*/*v*) solution at room temperature.

IR (Impack-410 Nicolet spectrometer, KBr, cm^−1^): 3510, 3334 (ν_NH_, ν_OH_); 3080, 2942 (ν_C-H_); 1588, 1540 (ν_C=Cring_ or ν_C=N_); 1190, 1036 (ν_C-O_, ν_S-O_), 632 (ν_C-Br_). ^1^H NMR (Bruker Avance 500 MHz, *d*_6_-DMSO): 8.34 (1H, *d*, *J* =1.0Hz, Ar), 8.27 (1H, *s*, Ar), 6.51 (1H, *s*, Ar), 4.22 (3H, *s*, N-CH3); 3.69 (2H, *t*, *J* = 5.5Hz); 3.45 (2H, *q*, *J* = 5.5Hz), 7.34 (NH).

## Refinement   

Crystal data, data collection and structure refinement details are summarized in Table 2 ▸. H atoms for N18, O21, O23, O24, O25 and O26 were located in difference Fourier maps. The coordinates of H21 and H26 were refined freely, while the other H atoms were refined as riding. All C-bound H atoms were placed at idealized positions and refined as riding, with C—H distances of 0.95 (aromatic), 0.99 (methyl­ene) and 0.98 Å (meth­yl). For most H atoms, *U*_iso_(H) values were assigned as 1.5*U*_eq_ of the parent atoms (1.2*U*_eq_ for H2, H4, H10, H18, H19*A*/*B* and H20*A*/*B*).

## Supplementary Material

Crystal structure: contains datablock(s) I. DOI: 10.1107/S2056989016012408/is5459sup1.cif

Structure factors: contains datablock(s) I. DOI: 10.1107/S2056989016012408/is5459Isup2.hkl

CCDC reference: 1497073

Additional supporting information: crystallographic information; 3D view; checkCIF report

## Figures and Tables

**Table 1:** Hydrogen-bond geometry (Å, °)

*D*—H⋯*A*	*D*—H	H⋯*A*	*D*⋯*A*	*D*—H⋯*A*
O21—H21⋯O17^i^	0.83 (3)	1.89 (3)	2.707 (2)	170 (3)
C11—H11*B*⋯Br16^ii^	0.98	3.02	3.987 (2)	171
C19—H19*A*⋯O13^ii^	0.99	2.59	3.360 (3)	134
N18—H18⋯O25^iii^	0.88	2.58	3.422 (2)	159
O23—H23*A*⋯O14^iv^	0.92	2.09	2.971 (2)	161
O23—H23*B*⋯O21^v^	0.91	1.72	2.630 (2)	172
O24—H24*A*⋯O13^ii^	0.90	1.90	2.772 (2)	162
O24—H24*B*⋯O17^vi^	0.90	1.83	2.714 (2)	165
O25—H25*A*⋯O15^vii^	0.92	2.16	2.826 (2)	129
O25—H25*B*⋯O26	0.91	1.86	2.755 (2)	165
O26—H26⋯O14^ii^	0.76 (3)	2.03 (3)	2.783 (2)	175 (3)

Symmetry codes: (i) 
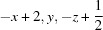
; (ii) 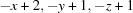; (iii) 
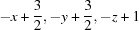
; (iv) 
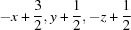
; (v) 
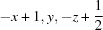
; (vi) 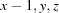; (vii) 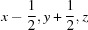.

**Table 2:** Experimental details

Crystal data	
Chemical formula	[Ni(H_2_O)_6_](C_12_H_12_BrN_2_O_5_S)_2_·H_2_O
*M*_r_	937.23
Crystal system, space group	Monoclinic, *C*2/*c*
Temperature (K)	100
*a*, *b*, *c* (Å)	8.7315 (4), 27.4581 (13), 13.7943 (6)
β (°)	94.061 (4)
*V* (Å^3^)	3298.9 (3)
*Z*	4
Radiation type	Mo *K*α
μ (mm^−1^)	3.22
Crystal size (mm)	0.4 × 0.2 × 0.1

Data collection	
Diffractometer	Agilent SuperNova (single source at offset, Eos detector)
Absorption correction	Multi-scan (*CrysAlis PRO*; Rigaku Oxford Diffraction, 2015 ▸)
*T*_min_, *T*_max_	0.546, 0.725
No. of measured, independent and observed [*I* > 2σ(*I*)] reflections	9171, 3372, 3041
*R*_int_	0.020
(sin θ/λ)_max_ (Å^−1^)	0.625

Refinement	
*R*[*F* ^2^ > 2σ(*F* ^2^)], *wR*(*F* ^2^), *S*	0.024, 0.056, 1.08
No. of reflections	3372
No. of parameters	235
H-atom treatment	H atoms treated by a mixture of independent and constrained refinement
Δρ_max_, Δρ_min_ (e Å^−3^)	0.41, −0.49

Computer programs: *CrysAlis PRO* (Rigaku Oxford Diffraction, 2015 ▸), *SHELXS97* (Sheldrick, 2008 ▸), *SHELXL2014* (Sheldrick, 2015 ▸) and *OLEX2* (Dolomanov *et al.*, 2009 ▸).

## supplementary crystallographic information

### Crystal data

**Table:**

[Ni(H_2_O)_6_](C_12_H_12_BrN_2_O_5_S)_2_·H_2_O	*F*(000) = 1904
*M**_r_* = 937.23	*D*_x_ = 1.887 Mg m^−^^3^
Monoclinic, *C*2/*c*	Mo *K*α radiation, λ = 0.71073 Å
*a* = 8.7315 (4) Å	Cell parameters from 5359 reflections
*b* = 27.4581 (13) Å	θ = 2.8–29.0°
*c* = 13.7943 (6) Å	µ = 3.22 mm^−^^1^
β = 94.061 (4)°	*T* = 100 K
*V* = 3298.9 (3) Å^3^	Plate, orange
*Z* = 4	0.4 × 0.2 × 0.1 mm

### Data collection

**Table:**

Agilent SuperNova (single source at offset, Eos detector) diffractometer	3372 independent reflections
Radiation source: micro-focus sealed X-ray tube, SuperNova (Mo) X-ray Source	3041 reflections with *I* > 2σ(*I*)
Mirror monochromator	*R*_int_ = 0.020
Detector resolution: 15.9631 pixels mm^-1^	θ_max_ = 26.4°, θ_min_ = 2.5°
ω scans	*h* = −10→10
Absorption correction: multi-scan (*CrysAlis PRO*; Rigaku Oxford Diffraction, 2015)	*k* = −34→32
*T*_min_ = 0.546, *T*_max_ = 0.725	*l* = −12→17
9171 measured reflections	

### Refinement

**Table:**

Refinement on *F*^2^	Primary atom site location: structure-invariant direct methods
Least-squares matrix: full	Hydrogen site location: mixed
*R*[*F*^2^ > 2σ(*F*^2^)] = 0.024	H atoms treated by a mixture of independent and constrained refinement
*wR*(*F*^2^) = 0.056	*w* = 1/[σ^2^(*F*_o_^2^) + (0.0207*P*)^2^ + 5.2045*P*] where *P* = (*F*_o_^2^ + 2*F*_c_^2^)/3
*S* = 1.08	(Δ/σ)_max_ = 0.002
3372 reflections	Δρ_max_ = 0.41 e Å^−^^3^
235 parameters	Δρ_min_ = −0.49 e Å^−^^3^
0 restraints	

### Special details

**Table:**

Geometry. All esds (except the esd in the dihedral angle between two l.s. planes) are estimated using the full covariance matrix. The cell esds are taken into account individually in the estimation of esds in distances, angles and torsion angles; correlations between esds in cell parameters are only used when they are defined by crystal symmetry. An approximate (isotropic) treatment of cell esds is used for estimating esds involving l.s. planes.

### Fractional atomic coordinates and isotropic or equivalent isotropic displacement parameters (Å^2^)

**Table:**

	*x*	*y*	*z*	*U*_iso_*/*U*_eq_	
N1	0.90237 (18)	0.43668 (6)	0.37155 (11)	0.0110 (3)	
C2	0.9801 (2)	0.39411 (8)	0.37514 (14)	0.0132 (4)	
H2	0.9254	0.3642	0.3739	0.016*	
C3	1.1379 (2)	0.39384 (8)	0.38056 (14)	0.0131 (4)	
C4	1.2182 (2)	0.43770 (8)	0.38321 (13)	0.0134 (4)	
H4	1.3273	0.4374	0.3877	0.016*	
C5	1.1403 (2)	0.48189 (8)	0.37930 (13)	0.0108 (4)	
C6	0.9750 (2)	0.48132 (8)	0.37471 (13)	0.0104 (4)	
C7	1.2125 (2)	0.52820 (8)	0.38177 (14)	0.0119 (4)	
C8	1.1373 (2)	0.57248 (8)	0.38109 (13)	0.0122 (4)	
C9	0.9683 (2)	0.56934 (8)	0.37556 (13)	0.0110 (4)	
C10	0.8918 (2)	0.52466 (8)	0.37301 (13)	0.0113 (4)	
H10	0.7828	0.5238	0.3701	0.014*	
C11	0.7332 (2)	0.43449 (8)	0.36504 (14)	0.0128 (4)	
H11A	0.6923	0.4543	0.3101	0.019*	
H11B	0.6950	0.4470	0.4253	0.019*	
H11C	0.6999	0.4006	0.3553	0.019*	
S12	1.23734 (6)	0.33760 (2)	0.37750 (4)	0.01589 (12)	
O13	1.3871 (2)	0.34725 (7)	0.42443 (13)	0.0360 (5)	
O14	1.24175 (17)	0.32666 (6)	0.27422 (10)	0.0200 (3)	
O15	1.1481 (2)	0.30278 (6)	0.42858 (12)	0.0323 (4)	
Br16	1.42949 (2)	0.53172 (2)	0.38060 (2)	0.01722 (7)	
O17	1.19947 (16)	0.61482 (5)	0.38363 (10)	0.0154 (3)	
N18	0.89569 (19)	0.61215 (7)	0.37205 (12)	0.0136 (4)	
H18	0.9506	0.6391	0.3730	0.016*	
C19	0.7298 (2)	0.61683 (8)	0.35993 (15)	0.0139 (4)	
H19A	0.6846	0.6070	0.4208	0.017*	
H19B	0.6892	0.5947	0.3075	0.017*	
C20	0.6831 (2)	0.66848 (8)	0.33499 (15)	0.0162 (4)	
H20A	0.5699	0.6704	0.3248	0.019*	
H20B	0.7159	0.6902	0.3899	0.019*	
O21	0.75070 (19)	0.68455 (6)	0.24924 (11)	0.0229 (4)	
H21	0.760 (3)	0.6610 (11)	0.213 (2)	0.034*	
Ni22	0.2500	0.7500	0.5000	0.01747 (10)	
O23	0.35588 (18)	0.75193 (6)	0.37316 (12)	0.0273 (4)	
H23A	0.3304	0.7799	0.3392	0.041*	
H23B	0.3270	0.7270	0.3322	0.041*	
O24	0.31091 (17)	0.67782 (6)	0.52289 (12)	0.0237 (4)	
H24A	0.4136	0.6748	0.5315	0.036*	
H24B	0.2816	0.6604	0.4695	0.036*	
O25	0.44976 (16)	0.77588 (5)	0.57273 (12)	0.0196 (3)	
H25A	0.5337	0.7662	0.5414	0.029*	
H25B	0.4680	0.7661	0.6357	0.029*	
O26	0.5000	0.73024 (9)	0.7500	0.0218 (5)	
H26	0.570 (3)	0.7142 (10)	0.746 (2)	0.033*	

### Atomic displacement parameters (Å^2^)

**Table:**

	*U*^11^	*U*^22^	*U*^33^	*U*^12^	*U*^13^	*U*^23^
N1	0.0109 (8)	0.0116 (9)	0.0102 (8)	0.0017 (7)	0.0001 (6)	−0.0009 (7)
C2	0.0169 (10)	0.0120 (10)	0.0106 (9)	0.0023 (8)	−0.0005 (8)	0.0001 (8)
C3	0.0151 (10)	0.0137 (11)	0.0102 (9)	0.0061 (8)	0.0001 (7)	−0.0021 (8)
C4	0.0125 (10)	0.0178 (11)	0.0098 (9)	0.0049 (8)	0.0007 (7)	−0.0004 (8)
C5	0.0103 (9)	0.0153 (11)	0.0070 (8)	0.0009 (8)	0.0010 (7)	−0.0001 (8)
C6	0.0118 (9)	0.0132 (11)	0.0064 (8)	−0.0002 (8)	0.0014 (7)	−0.0001 (8)
C7	0.0075 (9)	0.0184 (11)	0.0099 (9)	0.0003 (8)	0.0009 (7)	0.0005 (8)
C8	0.0109 (10)	0.0178 (11)	0.0078 (9)	−0.0010 (8)	0.0003 (7)	0.0001 (8)
C9	0.0111 (10)	0.0142 (11)	0.0077 (9)	0.0018 (8)	0.0003 (7)	−0.0011 (8)
C10	0.0079 (9)	0.0150 (11)	0.0111 (9)	0.0017 (8)	0.0007 (7)	0.0001 (8)
C11	0.0079 (9)	0.0140 (11)	0.0166 (10)	0.0003 (8)	0.0005 (7)	0.0003 (8)
S12	0.0176 (3)	0.0156 (3)	0.0141 (2)	0.0096 (2)	−0.00095 (19)	−0.0023 (2)
O13	0.0268 (9)	0.0349 (11)	0.0431 (11)	0.0212 (8)	−0.0198 (8)	−0.0210 (9)
O14	0.0243 (8)	0.0199 (9)	0.0156 (7)	0.0105 (7)	0.0008 (6)	−0.0038 (6)
O15	0.0481 (11)	0.0194 (9)	0.0315 (9)	0.0170 (8)	0.0186 (8)	0.0112 (8)
Br16	0.00740 (10)	0.02418 (13)	0.02029 (11)	0.00080 (8)	0.00253 (7)	0.00294 (9)
O17	0.0144 (7)	0.0140 (8)	0.0177 (7)	−0.0023 (6)	−0.0002 (6)	0.0003 (6)
N18	0.0113 (8)	0.0107 (9)	0.0189 (9)	−0.0004 (7)	0.0006 (7)	0.0002 (7)
C19	0.0094 (10)	0.0138 (11)	0.0184 (10)	0.0013 (8)	0.0001 (8)	0.0003 (9)
C20	0.0162 (10)	0.0160 (11)	0.0163 (10)	0.0034 (9)	−0.0004 (8)	−0.0003 (9)
O21	0.0355 (9)	0.0159 (9)	0.0179 (8)	0.0045 (7)	0.0053 (7)	0.0017 (7)
Ni22	0.00750 (18)	0.0097 (2)	0.0349 (2)	−0.00015 (14)	−0.00080 (16)	−0.00713 (17)
O23	0.0182 (8)	0.0217 (9)	0.0422 (10)	−0.0036 (7)	0.0036 (7)	−0.0123 (8)
O24	0.0136 (7)	0.0140 (8)	0.0422 (10)	0.0032 (6)	−0.0073 (7)	−0.0106 (7)
O25	0.0107 (7)	0.0148 (8)	0.0331 (9)	−0.0003 (6)	0.0004 (6)	−0.0042 (7)
O26	0.0133 (11)	0.0133 (12)	0.0403 (14)	0.000	0.0117 (10)	0.000

### Geometric parameters (Å, º)

**Table:**

N1—C2	1.351 (3)	S12—O15	1.4474 (18)
N1—C6	1.380 (3)	N18—H18	0.8806
N1—C11	1.475 (2)	N18—C19	1.452 (2)
C2—H2	0.9500	C19—H19A	0.9900
C2—C3	1.374 (3)	C19—H19B	0.9900
C3—C4	1.393 (3)	C19—C20	1.509 (3)
C3—S12	1.774 (2)	C20—H20A	0.9900
C4—H4	0.9500	C20—H20B	0.9900
C4—C5	1.390 (3)	C20—O21	1.429 (3)
C5—C6	1.440 (3)	O21—H21	0.83 (3)
C5—C7	1.418 (3)	Ni22—O23^i^	2.0366 (17)
C6—C10	1.394 (3)	Ni22—O23	2.0366 (17)
C7—C8	1.381 (3)	Ni22—O24	2.0704 (15)
C7—Br16	1.8983 (19)	Ni22—O24^i^	2.0704 (15)
C8—C9	1.474 (3)	Ni22—O25	2.0750 (14)
C8—O17	1.283 (3)	Ni22—O25^i^	2.0750 (14)
C9—C10	1.397 (3)	O23—H23A	0.9191
C9—N18	1.335 (3)	O23—H23B	0.9115
C10—H10	0.9500	O24—H24A	0.9003
C11—H11A	0.9800	O24—H24B	0.9001
C11—H11B	0.9800	O25—H25A	0.9163
C11—H11C	0.9800	O25—H25B	0.9128
S12—O13	1.4420 (17)	O26—H26	0.76 (3)
S12—O14	1.4592 (15)		
			
C2—N1—C6	122.61 (17)	O15—S12—O14	113.06 (10)
C2—N1—C11	117.74 (18)	C9—N18—H18	118.8
C6—N1—C11	119.64 (17)	C9—N18—C19	123.35 (18)
N1—C2—H2	119.8	C19—N18—H18	117.7
N1—C2—C3	120.4 (2)	N18—C19—H19A	109.4
C3—C2—H2	119.8	N18—C19—H19B	109.4
C2—C3—C4	119.84 (19)	N18—C19—C20	111.15 (17)
C2—C3—S12	119.61 (17)	H19A—C19—H19B	108.0
C4—C3—S12	120.46 (15)	C20—C19—H19A	109.4
C3—C4—H4	119.7	C20—C19—H19B	109.4
C5—C4—C3	120.66 (19)	C19—C20—H20A	109.4
C5—C4—H4	119.7	C19—C20—H20B	109.4
C4—C5—C6	118.55 (19)	H20A—C20—H20B	108.0
C4—C5—C7	124.51 (18)	O21—C20—C19	111.00 (17)
C7—C5—C6	116.92 (18)	O21—C20—H20A	109.4
N1—C6—C5	117.92 (18)	O21—C20—H20B	109.4
N1—C6—C10	121.32 (18)	C20—O21—H21	109 (2)
C10—C6—C5	120.76 (19)	O23^i^—Ni22—O23	180.00 (4)
C5—C7—Br16	119.16 (15)	O23^i^—Ni22—O24	88.36 (7)
C8—C7—C5	125.37 (18)	O23—Ni22—O24^i^	88.36 (7)
C8—C7—Br16	115.42 (15)	O23—Ni22—O24	91.64 (7)
C7—C8—C9	114.98 (19)	O23^i^—Ni22—O24^i^	91.64 (7)
O17—C8—C7	126.71 (18)	O23^i^—Ni22—O25^i^	89.39 (6)
O17—C8—C9	118.31 (18)	O23—Ni22—O25	89.39 (6)
C10—C9—C8	121.87 (19)	O23^i^—Ni22—O25	90.61 (6)
N18—C9—C8	114.94 (18)	O23—Ni22—O25^i^	90.61 (6)
N18—C9—C10	123.18 (18)	O24—Ni22—O24^i^	180.0
C6—C10—C9	120.10 (18)	O24^i^—Ni22—O25	86.79 (6)
C6—C10—H10	120.0	O24—Ni22—O25	93.21 (6)
C9—C10—H10	120.0	O24—Ni22—O25^i^	86.79 (6)
N1—C11—H11A	109.5	O24^i^—Ni22—O25^i^	93.21 (6)
N1—C11—H11B	109.5	O25^i^—Ni22—O25	180.0
N1—C11—H11C	109.5	Ni22—O23—H23A	110.6
H11A—C11—H11B	109.5	Ni22—O23—H23B	113.0
H11A—C11—H11C	109.5	H23A—O23—H23B	105.3
H11B—C11—H11C	109.5	Ni22—O24—H24A	110.6
O13—S12—C3	105.03 (10)	Ni22—O24—H24B	109.2
O13—S12—O14	112.98 (10)	H24A—O24—H24B	106.3
O13—S12—O15	113.89 (12)	Ni22—O25—H25A	110.3
O14—S12—C3	104.43 (9)	Ni22—O25—H25B	116.3
O15—S12—C3	106.39 (10)	H25A—O25—H25B	105.9
			
N1—C2—C3—C4	0.5 (3)	C6—N1—C2—C3	−1.1 (3)
N1—C2—C3—S12	−176.06 (14)	C6—C5—C7—C8	0.7 (3)
N1—C6—C10—C9	−179.52 (17)	C6—C5—C7—Br16	−176.56 (13)
C2—N1—C6—C5	1.8 (3)	C7—C5—C6—N1	179.55 (16)
C2—N1—C6—C10	−178.59 (17)	C7—C5—C6—C10	−0.1 (3)
C2—C3—C4—C5	−0.7 (3)	C7—C8—C9—C10	1.1 (3)
C2—C3—S12—O13	−156.31 (17)	C7—C8—C9—N18	−178.14 (17)
C2—C3—S12—O14	84.58 (17)	C8—C9—C10—C6	−0.6 (3)
C2—C3—S12—O15	−35.24 (19)	C8—C9—N18—C19	175.77 (17)
C3—C4—C5—C6	1.4 (3)	C9—N18—C19—C20	−166.55 (18)
C3—C4—C5—C7	179.87 (18)	C10—C9—N18—C19	−3.5 (3)
C4—C3—S12—O13	27.10 (19)	C11—N1—C2—C3	179.54 (17)
C4—C3—S12—O14	−92.01 (17)	C11—N1—C6—C5	−178.90 (16)
C4—C3—S12—O15	148.17 (17)	C11—N1—C6—C10	0.8 (3)
C4—C5—C6—N1	−1.9 (3)	S12—C3—C4—C5	175.84 (14)
C4—C5—C6—C10	178.47 (17)	Br16—C7—C8—C9	176.19 (13)
C4—C5—C7—C8	−177.81 (18)	Br16—C7—C8—O17	−3.0 (3)
C4—C5—C7—Br16	5.0 (3)	O17—C8—C9—C10	−179.67 (17)
C5—C6—C10—C9	0.1 (3)	O17—C8—C9—N18	1.1 (3)
C5—C7—C8—C9	−1.1 (3)	N18—C9—C10—C6	178.53 (17)
C5—C7—C8—O17	179.72 (18)	N18—C19—C20—O21	57.0 (2)

Symmetry code: (i) −*x*+1/2, −*y*+3/2, −*z*+1.

### Hydrogen-bond geometry (Å, º)

**Table:**

*D*—H···*A*	*D*—H	H···*A*	*D*···*A*	*D*—H···*A*
O21—H21···O17^ii^	0.83 (3)	1.89 (3)	2.707 (2)	170 (3)
C11—H11*B*···Br16^iii^	0.98	3.02	3.987 (2)	171
C19—H19*A*···O13^iii^	0.99	2.59	3.360 (3)	134
N18—H18···O25^iv^	0.88	2.58	3.422 (2)	159
O23—H23*A*···O14^v^	0.92	2.09	2.971 (2)	161
O23—H23*B*···O21^vi^	0.91	1.72	2.630 (2)	172
O24—H24*A*···O13^iii^	0.90	1.90	2.772 (2)	162
O24—H24*B*···O17^vii^	0.90	1.83	2.714 (2)	165
O25—H25*A*···O15^viii^	0.92	2.16	2.826 (2)	129
O25—H25*B*···O26	0.91	1.86	2.755 (2)	165
O26—H26···O14^iii^	0.76 (3)	2.03 (3)	2.783 (2)	175 (3)

Symmetry codes: (ii) −*x*+2, *y*, −*z*+1/2; (iii) −*x*+2, −*y*+1, −*z*+1; (iv) −*x*+3/2, −*y*+3/2, −*z*+1; (v) −*x*+3/2, *y*+1/2, −*z*+1/2; (vi) −*x*+1, *y*, −*z*+1/2; (vii) *x*−1, *y*, *z*; (viii) *x*−1/2, *y*+1/2, *z*.
